# Chronic and Occult Hepatitis B Virus Infection in Pregnant Women in Botswana

**DOI:** 10.3390/genes9050259

**Published:** 2018-05-17

**Authors:** Tshepiso Mbangiwa, Ishmael Kasvosve, Motswedi Anderson, Prisca K. Thami, Wonderful T. Choga, Austen Needleman, Bonolo B. Phinius, Sikhulile Moyo, Melvin Leteane, Jean Leidner, Jason T. Blackard, Gloria Mayondi, Betsy Kammerer, Rosemary M. Musonda, Max Essex, Shahin Lockman, Simani Gaseitsiwe

**Affiliations:** 1 Botswana Harvard AIDS Institute Partnership, Gaborone BO320, Botswana; mbangiwat@gmail.com (T.M.); manderson@bhp.org.bw (M.A.); pthami@bhp.org.bw (P.K.T.); wtchoga@gmail.com (W.T.C.); bphinius@gmail.com (B.B.P.); smoyo@bhp.org.bw (S.M.); gmayondi@bhp.org.bw (G.M.); rmusonda@bhp.org.bw (R.M.M.); messex@hsph.harvard.edu (M.E.); shahin.lockman@gmail.com (S.L.); 2 Department of Medical Laboratory Sciences, Faculty of Health Sciences, University of Botswana, Gaborone 0022, Botswana; kasvosvei@ub.ac.bw; 3 Department of Biological Sciences, Faculty of Science, University of Botswana, Gaborone 0022, Botswana; LETEANEM@mopipi.ub.bw; 4 Harvard College, Cambridge, MA 02138, USA; aneedleman@college.harvard.edu; 5 Department of Immunology and Infectious Diseases, Harvard T.H. Chan School of Public Health, Boston, MA 02115, USA; 6 Goodtables Data Consulting, Norman, OK 73019, USA; jeanleid@gmail.com; 7 University of Cincinnati College of Medicine, Cincinnati, OH 45627, USA; blackajt@ucmail.uc.edu; 8 Department of Psychiatry, Boston Children’s Hospital, Boston, MA 02115, USA; Betsy.Kammerer@childrens.harvard.edu; 9 Brigham and Women’s Hospital, Boston, MA 02115, USA

**Keywords:** pregnant women, hepatitis B virus (HBV), human immunodeficiency virus (HIV), Botswana

## Abstract

The hepatitis B virus (HBV) is a global problem; however, the burden of HBV infection in pregnant women in Botswana is unknown. We sought to determine the prevalence of chronic and occult HBV infection in human immunodeficiency virus (HIV)-infected and -uninfected pregnant women in Botswana. Samples from 752 pregnant women were tested for hepatitis B surface antigen (HBsAg), and HBsAg-positive samples were tested for hepatitis B e antigen (HBeAg) and HBV DNA load. Samples that were HBsAg negative were screened for occult HBV infection by determining the HBV DNA load. HBV genotypes were determined based on a 415-base-pair fragment of the surface gene. Among the 752 women tested during pregnancy or early postpartum, 16 (2.1%) (95% confidence interval (CI): 2.0–2.2) were HBsAg-positive. The prevalence of chronic HBV infection was higher (3.1%) among HIV-infected (95% CI: 3.0–3.2) compared with HIV-uninfected women (1.1%) (95% CI: 1.07–1.1, *p* = 0.057). Among the 622 HBsAg-negative women, the prevalence of occult HBV infection was 6.6% (95% CI: 6.5–6.7). Three of thirteen HBsAg-positive participants were HBeAg-positive, and all were HIV-negative. Of the 11 maternal samples successfully genotyped, five (45.5%) were genotype D3, five (45.5%) were genotype A1, and one was genotype E (9%). Low and similar proportions of HIV-infected and -uninfected pregnant women in Botswana had occult or chronic HBV infection. We identified a subset of HIV-negative pregnant women who had high HBV DNA levels and were HBeAg-positive, and thus likely to transmit HBV to their infants.

## 1. Introduction

Viral hepatitis is a major cause of morbidity and mortality, and it is ranked as the seventh-leading cause of mortality in the world [1]. Hepatitis B virus (HBV) infection causes 70% of the viral hepatitis-related mortality [2]. Global estimates suggest that 3.6% of the world’s population is infected with HBV [3], with the highest burden in Asia and sub-Saharan Africa [4,5]. Human immunodeficiency virus (HIV) and HBV are significant public health threats in sub-Saharan Africa. It is well documented that HIV/HBV coinfection impacts the natural progression of HBV infection, resulting in increased HBV transmission and decreased host immune responses [6,7,8].

In most highly HBV-prevalent regions, the majority of HBV infections occur in early childhood, either through vertical or horizontal transmission [9]. HBV infection during early childhood is more likely to result in chronic HBV infection [10,11] compared to infection occurring later in life. While considerable focus has been made on reducing the vertical transmission of HIV in sub-Saharan Africa, research on the prevention of HBV vertical transmission in sub-Saharan Africa is quite limited. Therefore, there is a need to generate country-specific data to determine the rates of HBV infection in pregnant women and children and to evaluate if the intensification of HBV prevention strategies is needed within the country. 

Botswana, a southern African country with a population of approximately 2.3 million, has an intermediate prevalence of HBV [12]. The country also has one of the highest prevalence of HIV globally, at 18.5% [13]. There is scant data on HBV prevalence in Botswana, with most of the available data coming from HIV-infected cohorts in whom HBV prevalence ranges between 5% and 10% [4,14,15], including in HIV-infected pregnant women [16]. Data regarding HBV prevalence in HIV-uninfected pregnant women is nonexistent in Botswana. We sought to determine the prevalence of chronic and occult HBV infection in HIV-infected and -uninfected pregnant women in Botswana. 

## 2. Materials and Methods

### 2.1. Study Participants

We used stored samples and existing data from the completed Tshipidi study that has been described in detail previously [17]. Briefly, Tshipidi was a prospective cohort study that enrolled and followed 453 HIV-infected and 457 HIV-uninfected pregnant women and their live-born infants at two sites (the city of Gaborone and the village of Mochudi) in Botswana from 2010 to 2012. Mothers and their infants were followed to evaluate the effects of in utero exposure to maternal HIV or to antiretroviral drugs on neurodevelopment and child health outcomes at two years of age. For the current study, samples from all mothers at delivery (and also at enrollment for the chronic HBV-positive participants) were included, provided that the relevant stored sample(s) were still available. The delivery samples were drawn at least two months apart from the enrollment samples. 

Tshipidi study participants provided written informed consent for study participation. The study was done in accordance with the Declaration of Helsinki, and the research protocol was approved by the Health Research Development Committee at the Botswana Ministry of Health and Wellness (HRDC00524) and the Office of Human Research Administration at the Harvard T.H. Chan School of Public Health (18093). 

### 2.2. Hepatitis B Virus Serological Screening

Available Tshipidi plasma samples were tested for hepatitis B surface antigen (HBsAg) using a Murex HBsAg kit (Murex Biotech, Dartford, UK). The positive samples were repeated to validate their positive status using the Murex HBsAg kit. The samples that were positive for HBsAg were screened for hepatitis E antigen (HBeAg) using the Monolisa HBeAg-Ab PLUS kit (Bio-Rad, Hercules, CA, USA).

### 2.3. Hepatitis B Virus DNA Levels

Maternal samples were tested for HBV DNA levels. HBsAg positive samples were also tested at the enrollment visit. HBV DNA levels were quantified using the COBAS^®^ AmpliPrep COBAS^®^ Taqman^®^, HBV Test v.2.0 (Roche diagnostics, Mannheim, Germany) following the manufacturer’s instructions. The limit of detection was 20 IU/mL. HBV DNA suppression was defined as undetectable (not detected or ND) HBV DNA. 

### 2.4. DNA Extraction for Genotyping

DNA was extracted from 200 μL of plasma samples using the QiAmp DNA extraction kit according to the manufacturer’s protocol (QIAGEN, Hilden, Germany). An elution volume of 50 μL was used instead of the 200 μL recommended in the protocol to increase sample concentration. DNA extracts were either used for amplification directly or stored at −80 °C until use.

### 2.5. HBV Genotyping

Chronic HBV positive samples were genotyped using a previously published protocol [18]. Briefly, a 415-base-pair (bp) fragment of the surface gene was amplified using a seminested polymerase chain reaction (PCR) using the SuperScript™ III One-Step RT-PCR System with Platinum^®^ Taq High Fidelity (Life technologies, Waltham, MA, USA). Primers HBV381 (5’-GTTTAAATGTATACCCAAAGAC-3’; nt 840–861) and HBV840 (5’-TGCGGCGTTTTATCATCTTCCT-3’; nt 381–402) were used for the first round of PCR, with cycling conditions as previously described [4]. HBV801 (5’-CGACGGCATAAAGGGACTCAAG-3’: nt 801–822) and HBV381 were used for the second round [4]. A 2% agarose gel stained with ethidium bromide was used to view the PCR product. The PCR products were cleaned using the QIAquick^®^ PCR purification kit (QIAGEN, Mannheim, Germany). Primers HBV801 and HBV381 were used for the sequencing reactions. DNA was labeled using ABI BigDye^®^ v3.1 terminators (Applied Biosystems, Foster City, CA, USA) and purified using ZR DNA Sequencing Clean-up Kit™ (Zymo, Irvine, CA, USA) as per manufacturers’ protocol. The sequencing chromatograms were created using an ABI 3130xl genetic analyzer (Applied Biosystems). 

Chromatographs were edited using Sequencher version 5.0 [19] to generate consensus sequences. Nucleotide alignments were performed with Clustal X 2.1 [20]. Representative references of genotypes A–H were retrieved from GenBank and aligned with sequences from this study to determine genotypes. The National Center for Biotechnology Information (NCBI) GenBank accession numbers for sequences found in this study are MG977689, MG977690, MG977693, MG977694, MG977695, MG977696, MG977697, MG977698, MG977699, MG977700, and MG977701. Additional phylogenetic inference was performed using a Bayesian Markov chain Monte Carlo (MCMC) approach as implemented in the Bayesian evolutionary analysis by sampling trees (BEAST) version 1.8.4 program [21], with an uncorrelated log-normal relaxed molecular clock, general time-reversible model, and nucleotide site heterogeneity estimated using a gamma distribution. The MCMC analysis was run for a chain length of 100,000,000, and results were visualized to confirm adequate chain convergence with Tracer version 1.6 [22]. The effective sample size (ESS) was calculated for each parameter, and all ESS values were >500, indicating sufficient sampling. The maximum clade credibility tree was selected from the posterior tree distribution after a 10% burn-in using Tree Annotator version 1.8.4 [21] and visualized in FigTree version 1.4.3 [23]. Posterior probabilities >0.90 are noted as statistically significant, as described previously [24,25].

### 2.6. Data Analysis

Chronic HBV infection was defined as HBsAg positivity, while occult HBV infection was defined as HBsAg negativity, but with detectable HBV DNA. Data were reported using proportions, medians, and interquartile ranges (IQR). Categorical and continuous characteristics were compared by HBV status using Fisher’s exact test or Wilcoxon’s rank sum test, respectively. All data were analyzed using STATA v14.0 [26], and *p*-values <0.05 were considered statistically significant. 

## 3. Results 

**Figure 1:**
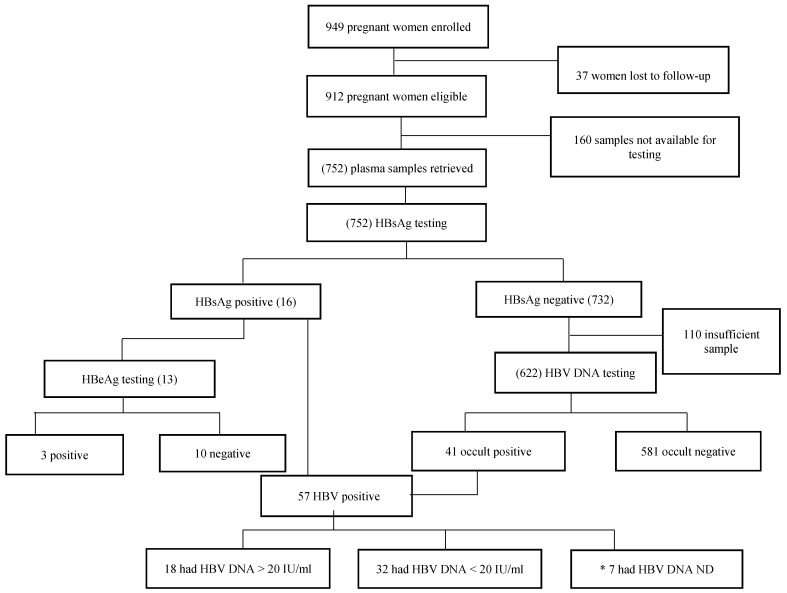
Screening algorithm showing the different tests that were performed and the results obtained. HBsAg: hepatitis B surface antigen, HBeAg: hepatitis B e antigen, HBV: hepatitis B virus, ND: not detectable, *: all these were HBsAg-positive.

Of 912 mothers that were enrolled in the parent cohort, 752 were screened for HBsAg (chronic HBV infection), and 622 out of the 752 were screened for occult HBV infection (i.e., HBsAg-negative with detectable HBV DNA). Samples from participants with positivity for the HBsAg results were tested for HBeAg, and the HBV-positives were genotyped as per Figure 1. 

Of the 912 women, 454 were HIV-infected and 458 HIV-uninfected. Of the 752 women, 384 are HIV-positive and 368 are HIV-negative. The median CD4+ T-cell counts of the HIV-positive maternal participants were 422 (313, 567), 460 (340, 511), 604 (322, 616), 418 (313, 563), and 481 (289, 628) for the chronic HBV-positive, occult HBV-positive, HBV-negative, and the total HBV-positive groups, respectively. The aspartate transaminase (AST), alanine transaminase (ALT), HIV status, HIV viral load, and other characteristics were not significantly different between the different groups (Table 1). 

**Figure 2:**
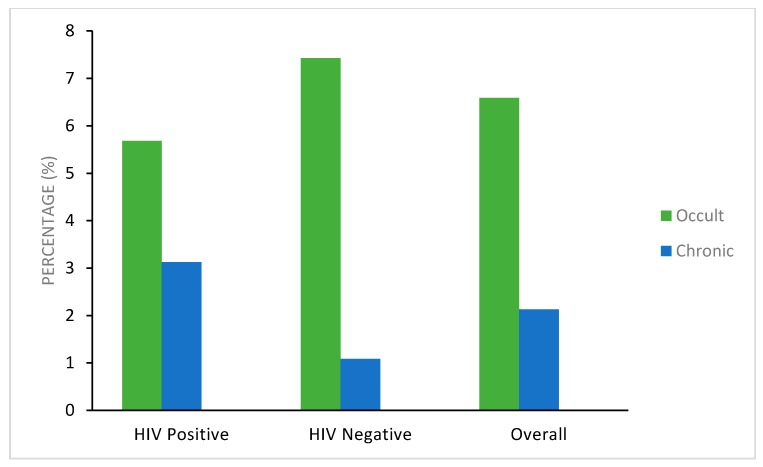
Prevalence of occult and chronic HBV infection amongst the human immunodeficiency virus (HIV) positive and HIV negative pregnant women.

The prevalence of HBV infection in pregnant women was 2.1% (16/752), and 6.6% (41/622) for chronic and occult HBV infection, respectively. Chronic HBV positivity was significantly higher in HIV-positive women (3.1%; 12 of 384) compared to HIV-negative women (1.1%; 4 of 368; *p* = 0.057), as shown in Figure 2. However, there was no difference in occult HBV infection in HIV-negative women as compared to HIV-positive women (24 (7.4%) of 323 versus 17 (5.7%) of 299 women, respectively (*p* = 0.38)).

HBsAg-positive women who were HIV-negative had HBV DNA levels between 396 IU/mL and > 1.70 × 10^8^ IU/mL, and three out of four were HBeAg. Of the 13 participants with chronic HBV infection, three were HBeAg-positive and all were HIV-negative. These three participants had HBV DNA levels > 1.7 × 10^8^ IU/L at both enrolment and delivery. The delivery samples were drawn at least two months apart from the enrollment samples. All 12 HIV-positive chronically infected participants had HBV DNA levels between < 20 IU/mL and 12,900 IU/mL. Of these women, 58.3% (7/12) were on Zidovudine (AZT) monotherapy and 41.7% (5/12) were on highly active antiretroviral therapy (HAART). 

We then evaluated the prevalence of HBV infections amongst HIV-positive participants with a CD4+ T-cell count of > 500 cells/mL and those with a CD4+ T cell count of ≤ 500 cells/mL. This was to determine if there is an association of HBV infection with CD4+ T-cell counts in HIV-infected participants.

Of the 384 tested for chronic HBV infection, 382 had available CD4+ T-cell count results, and of the 299 tested for occult HBV infection, 297 had available CD4+ T-cell count results. Amongst the 382 tested for chronic HBV infection, 129 had CD4+ T-cell counts of > 500 cells/mL and 253 had CD4+ T-cell counts of ≤ 500 cells/mL. Amongst the 129 with CD4+ T-cell counts of > 500 cells/mL, three were chronically HBV-infected, and out of the 253 with ≤ 500 cells/mL, nine were chronically HBV-infected, yielding a chronic HBV infection prevalence of 3/129 (2.33%) and 9/253 (3.56%), respectively. For the 297 tested for occult HBV infection, 96 had CD4+ T-cell counts of > 500 cells/mL and 201 had CD4+ T-cell counts of ≤ 500 cells/mL. Amongst the 96 with CD4+ T-cell counts of > 500 cells/mL, 10 had occult HBV infection, and of the 201 with CD4+ T-cell counts of ≤ 500 cells/mL, seven had occult HBV infection, yielding an occult HBV infection prevalence of 10/96 (10.4%) and 7/201 (3.48%), respectively. 

There was a significant difference in the prevalence of occult HBV infection in women with CD4+ T-cell counts of > 500 cells/mL compared to those with a CD4+ T-cell count of ≤ 500 cells/mL (*p*-value = 0.01). Of the participants that were on treatment (*n* = 267), 96 had a CD4+ T-cell count of > 500 cells/mL (three of whom were receiving unspecified anti retroviral therapy, ART) and 171 had a CD4+ T-cell count of ≤ 500 cells/mL (four of whom were receiving unspecified ART). There was no difference in the CD4+ T-cell counts in women taking AZT or HAART. 

**Figure 3:**
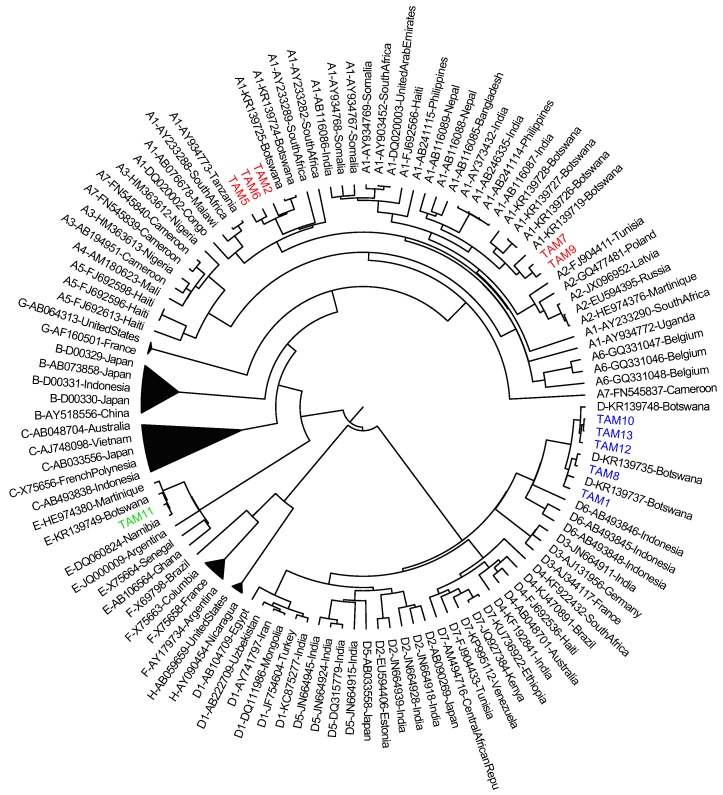
Bayesian phylogenetic analysis of S gene sequences (indicated by participant code (TAM number) compared to reference sequences (indicated by their subgenotypes, accession number, and country of origin). HBV genotype A sequences (*n* = 5) are shown in red, genotype D sequences (*n* = 5) in blue, and genotype E sequences (*n* = 1) in green.

Of the 16 chronic HBV infections, 11 (69%) samples were successfully genotyped (Table 2). Five samples (45.5%) were genotype D, five (45.5%) were genotype A, and one was genotype E (9%). Figure 3 shows the clustering of the different genotypes with the genotype references. All the sequences cluster with sequences from Botswana and other African countries. One genotype D sequence had a T131N immune escape mutation. 

## 4. Discussion

In this study, we present the largest analysis of both chronic and occult HBV infection prevalence in HIV-infected and HIV-uninfected pregnant women in Botswana, a sub-Saharan African country with high prevalence of HIV and an intermediate prevalence of HBV. The chronic and occult HBV infection prevalence in the pregnant women was 2.1% and 6.6%, respectively, and there was no difference between the prevalence rates in the HIV-positive and HIV-negative participants. The median age of the pregnant women was 27 years, and age did not seem to be a risk factor for HBV infection as there was no difference in ages of the women with HBV versus those without HBV. However, a recent study in Uganda reported higher rates of new HBV infections among HIV-infected patients, which were drastically reduced by initiation of combination antiretroviral therapy (cART) [27]. Other studies in Botswana have reported similar prevalence rates, although these studies included only HIV-infected individuals [4,16]. Higher prevalence rates have been reported by others in the region, ranging from 6.9% to 11.6% [28,29,30]. 

Occult HBV infection prevalence reported in this study is lower than the findings by Ryan et al., who reported a prevalence of 26.5% in HIV-positive patients in Botswana at baseline [31]. Although the results of our study show a lower prevalence of occult HBV infection compared to the prevalence found by Ryan et al. in HIV-infected treatment-naïve patients, our results are comparable with those from the same study after the participants had initiated cART (1.5%) [31]. 

HIV-positive women with chronic HBV infection had lower HBV DNA levels compared to the HIV-negative women at both enrollment and delivery visits. Three out of four HIV-negative women had positive HBeAg results and high HBV DNA levels. High HBV DNA and HBV E antigen (HBeAg) positivity are risk factors for HBV vertical transmission; therefore, there is a likelihood of passing on the infection as these women are not taking any anti-HBV drugs [32]. HBsAg positivity and non-occult infection has been linked to vertical transmission [33,34]. These findings are concerning, as HBV is not part of the antenatal screening tests done in Botswana. However, the vaccination of all babies against HVB is a strategy that the Botswana Ministry of Health and Wellness implemented in the early 2000s. Infants are given the first dose at birth, the second at one month of age, and the last at two months of age, thereby decreasing the chance of these children developing chronic or occult HBV infection. 

The low HBV DNA levels in the HIV-positive mothers could be a result of the cross-effect of the ART treatment that the women were receiving. In total, 75% of the women were taking ART drugs (HAART and AZT), and the remaining 25% did not have any documented ART, but had lower HBV DNA levels. Some of the HAART that these women were taking contain tenofovir (TDF), emtricitabine (FTC), and lamivudine (3TC), which have anti-HBV properties, and this could explain the low HBV DNA levels and HBeAg negativity in the HIV-positive individuals [35,36,37]. Tenofovir and lamivudine are used worldwide as first-line HBV treatments due to their high barrier to resistance and tolerability [38]. The “treat all” strategy was implemented in June 2016 in Botswana as per the handbook of the 2016 Botswana integrated HIV clinical care guidelines [39]. The first line “treat all” regimen contains Truvada (emtricitabine and tenofovir disoproxil fumarate) and dolutegravir (DTG). Thus, one would expect the rates of HBV and HIV transmission to decline in the coming years. 

The prevalence of the occult-infected participants was 10.4% in participants with CD4+ T-cell counts of >500 cells/mL and 3.48% in participants with CD4+ T-cell counts of ≤500 cells/mL (Table 3). This is contrary to what other studies have found. Several studies have shown that occult HBV infection is more prevalent in HIV-positive people with lower CD4+ T-cell counts as opposed to people with high CD4+ T-cell counts [40,41]. The low prevalence of occult HBV infection in the group with CD4+ T-cell counts of <500 cells/mL could be due to the protective effect of HAART, which might reduce the HBV DNA to undetectable levels (viral suppression) [35]. A CD4+ T-cell count cutoff of 500 cells/mL was used. This was to compare “typically” healthy HIV-positive patients with those that are “unhealthy” and investigate associations with ART treatment and type of HBV infection [42,43]. This has also been supported by the INSIGHT START study group [44]. 

The genotypes detected in this study are similar to those reported by other studies in the region [4,45]. Genotype A is more prevalent in Southern Africa, followed by D and then E. Anderson et al. reported a higher proportion of genotype A compared to other genotypes [4]. Genotypes A, D, and E are not linked to high levels of HBV vertical transmission, as are genotypes B and C, which are associated with high HBV DNA levels and HBeAg positivity [46]. However, our results indicate that in some cases, pregnant women with HBV genotypes not traditionally associated with vertical transmission do have high HBV DNA levels, which put them at risk of transmitting HBV. The T131N mutation that was found from our study is found on the “a” determinant region of the surface gene [47]. Computational analysis has revealed that T131N substitutions led to an insertion of an “extra” α-helix in the “a” determinant region [47]. This has been associated with exacerbations of chronic infections and the rescue of virion secretions [48,49]. 

Despite the limited sample size and volumes used in this study, the study highlights the need for testing pregnant women for HBV and for those testing positive to check their HBV DNA levels. HBV-positive pregnant women with high HBV DNA levels need to be considered for further HBV prevention of mother to child transmission (PMTCT) measures, as proposed by Wilson et al. [50]. We could not confirm whether the samples that tested HBsAg-positive were acute or chronic infections; however, most HBV infections in sub-Saharan Africa are acquired in early childhood, and the assumption made here was that these were chronic infections [10,51,52]. 

## Figures and Tables

**Table 1:** Association of clinical demographics with chronic versus occult hepatitis B virus (HBV) infection.

	All (*N* = 752)	Chronic HBV (*N* = 16)	Occult HBV (*N* = 41)	*p*-Value	Chronic or Occult HBV (*N* = 57)	HBV-Negative (*N* = 695)	*p*-Value
Media maternal age in years (*n* = 581) (Q1, Q3)	27 (24, 32)	29 (25, 33)	28 (23, 32)	0.58	27 (23, 31)	27 (23, 32)	0.24
HIV-positive women	384 * and 299 ^+^	12 (3.1%)	17 (5.7%)	0.057 ^a^	29 (7.6%)	355 (92.4%)	0.54
HIV-negative women	368 * and 323 ^+^	4 (1.1%)	24 (7.4%)	0.38 ^b^	28 (7.6%)	340 (92.3%)	
HIV viral load (copies/mL) (Q1, Q3)	1549.5 (400, 13,808)	508.5 (193, 3819)	1568 (395, 9635)	0.70	676 (400, 7762)	1585 (400, 14,125)	0.16
CD4+ T-cell count (cells/μL) (Q1, Q3)	422 (313–567)	460 (340, 511)	604 (322, 616)	0.14	481 (289, 628)	418 (313, 563)	0.25
Platelet count (Q1, Q3)	267 (216–311)	296 (211, 365)	272 (228, 371)	0.18	298 (233, 371)	265 (211, 309)	0.52
Hemoglobin (g/dL) (Q1, Q3)	11 (10–12)	12 (11, 12)	11 (9.0, 12)	0.19	12 (10, 13)	10.9 (9, 12)	1.00
ALT (U/L) (Q1, Q3)	13 (11, 19)	12 (10, 13)	14 (12, 19)	0.74	13 (10, 14)	13 (11, 17)	0.65
AST (U/L) (Q1, Q3)	23.4 (19, 30)	24 (18, 30)	22 (18, 30)	0.30	30 (21, 35)	23 (19, 30)	0.35
Total bilirubin (μmol/L) (Q1, Q3)	6 (4, 8)	4 (2.9, 5.7)	4.7 (4, 12)	0.10	4 (3, 6)	6 (4, 8)	1.00

HIV: Human immunodeficiency virus, ALT: Alanine transaminase, AST: Aspartate transaminase, Q1; first quartile, Q3: third quartile,*: totals for participants tested for chronic HBV infection, ^+^: totals for participants tested for occult HBV infection, ^a^: *p*-value for chronic infection in the HIV-positive women versus HIV-negative women, ^b^: *p*-value for occult infection in HIV-positive versus HIV-negative women.

**Table 2:** HBV DNA levels at enrollment and delivery, and HBeAg status for the 16 women with chronic HBV infection (HBsAg-positive).

Participant Code	HBV DNA at Enrollment (IU/L)	HBV DNA at Delivery (IU/L)	HBeAg at Delivery	HIV Status	ART Regimen	Genotype
TAM 1	NT	ND	NEG	POS	AZT	D
TAM 2	NT	ND	NEG	POS	AZT	A
TAM 5	8710	ND	NEG	POS	AZT	A
TAM 6	<20	ND	NEG	POS	CBV + NVP	A
TAM 7	<20	2250	NEG	POS	AZT	A
TAM 8	>1.70 × 10^8^	>1.70 × 10^8^	POS	NEG	NA	D
TAM 9	>1.70 × 10^8^	>1.70 × 10^8^	POS	NEG	NA	A
TAM 10	>1.70 × 10^8^	>1.70 × 10^8^	POS	NEG	NA	D
TAM 11	170	396	NT	NEG	NA	E
TAM 12	NT	12,900	NT	POS	AZT	D
TAM 13	NT	ND	NT	POS	NVP ± TRU	D
TAM 14	25	ND	NEG	POS	CBV + NVP	NA
TAM 15	24.8	<20	NEG	POS	ATR	NA
TAM 16	237	ND	NEG	POS	CBV + NVP	NA
TAM 17	82	166	NEG	POS	AZT	NA
TAM 18	ND	2300	NT	POS	AZT	NA

AZT: zidivodine; NA: not applicable; NT: not tested; ND: target not detected; HAART: highly active antiretroviral therapy. The women were on different HAART combinations, including Combivir (CBV) + Nevirapine (NVP) and Truvada (TRU) + Nevirapine, as well as Atripla (ATR) alone.). NEG: negative; POS: positive; TAM: participant unique code.

**Table 3:** Prevalence of different HBV infections and antiretroviral therapy (ART) status amongst HIV-positive participants with low and high CD4+ T-cell counts.

CD4+ T-Cell Count (cells/mL)	Chronic HBV infection (*n* = 382) ^a^	Occult HBV infection (*n* = 297) ^b^	AZT (*n* = 267)	HAART (*n* = 267)
> 500	3/129 (2.33%)	10/96 (10.4%)	78/96 (81.3%)	15/96 (15.6%)
≤ 500	9/253 (3.56%)	7/201 (3.48%)	124/171 (72.5%)	43/171 (25.1%)
*p*-value	0.511	0.01	0.110	0.07

^a^: number of participants with available CD4+ T-cell counts tested for chronic HBV infection, ^b^: number of participants with available CD4+ T-cell counts tested for occult HBV infection, AZT: Zidovudine, HAART: Highly active antiretroviral therapy.
